# Paper trials: a qualitative study exploring the place of portfolios in making revalidation recommendations for Responsible Officers

**DOI:** 10.1186/s12909-016-0592-6

**Published:** 2016-02-17

**Authors:** Daniel S. Furmedge, Ann Griffin, Catherine O’Keeffe, Anju Verma, Laura-Jane Smith, Deborah Gill

**Affiliations:** University College London Medical School, 74 Huntley Street, London, WC1E 6AU UK; Health Education North West London, London, UK

**Keywords:** Revalidation, Appraisal, Quality, Learning tools, Assessment, Clinical governance, Reflection, Portfolios

## Abstract

**Background:**

A portfolio of supporting information (SI) reflecting a doctor’s entire medical practice is now a central aspect of UK appraisal for revalidation. Medical revalidation, introduced in 2012, is an assessment of a doctor’s competence and passing results in a five yearly license to practice medicine. It assesses of a doctor’s professional development, workplace performance and reflection and aims to provide assurance that doctors are up-to-date and fit to practice. The dominant assessment mechanism is a portfolio. The content of the revalidation portfolio has been increasingly prescribed and the assessment of the SI is a fundamental aspect of the appraisal process which ultimately allows Responsible Officers (ROs) to make recommendations on revalidation. ROs, themselves doctors, were the first to undergo UK revalidation. This qualitative study explored the perceptions of ROs and their appraisers about the use of this portfolio of evidence in a summative revalidation appraisal.

**Methods:**

28 purposefully sampled London ROs were interviewed following their revalidation appraisal and 17 of their appraisers participated in focus groups and interviews. Thematic analysis was used to identify commonalities and differences of experience.

**Results:**

SI was mostly easy to provide but there were challenges in gathering certain aspects. ROs did not understand in what quantities they should supply SI or what it should look like. Appraisers were concerned about making robust judgements based on the evidence supplied. A lack of reflection from the process of collating SI and preparing for appraisal was noted and learning came more from the appraisal interview itself.

**Conclusions:**

More explicit guidance must be available to both appraisee and appraiser about what SI is required, how much, how it should be used and, how it will be assessed. The role of SI in professional learning and revalidation must be clarified and further empirical research is required to examine how best to use this evidence to make judgments as part of this type of appraisal.

## Background

The revalidation of United Kingdom (UK) doctors began in 2012 prompted by a need to increase public confidence in the regulation of medical profession [1]. Medical revalidation is the process whereby doctors are proactively assessed to ensure that they are up-to-date and fit to practice, and if deemed so, they are granted a license to practice for five years providing they remain in good standing. Revalidation is largely dependent on the production of a portfolio with must contain core, standardized supporting information (SI), successful annual appraisals based on the General Medical Council’s (GMC) *Good Medical Practice* framework (GMP), and the absence of significant concerns [2]. *Good Medical Practice* necessitates that doctors provide supporting information and reflection across four domains:

1. Knowledge, skills, and performance
2. Safety and quality
3. Communication, partnership, and teamwork
4. Maintaining trust

Within each domain, specific items of supporting information must be provided [3]. These must span the full breadth of practice of the doctor and must be reflected upon. However, guidance does not explicitly describe what represents quality in the SI or the reflective writing that accompanies it. Mandatory items which must be provided as a minimum are listed in Table 1 [4].

**Table 1:** Mandatory SI items comprising the minimum dataset

• Multisource feedback (MSF) patient survey • MSF from colleagues • Complaints and compliments • Quality improvement, serious untoward incidents and two case reviews • Continuing professional development activities (50 credits)	

Although maintaining a reflective portfolio and providing SI is now an established part of postgraduate training, this requirement is new for many senior doctors. The use of a portfolio containing SI is arguably part of a wider movement of competency based training and assessment in medical education but is as yet largely under-explored in the context of assessment in continuous professional development and re-licensing [5]. The literature from undergraduate and postgraduate training identifies that when portfolios are used to make summative judgements, this influences the choice of SI, prioritising documentation which portrays competence [5, 6].

Reflective practice has become an accepted organizing framework for contemporary professional practice and reflection on practice is now a mandatory aspect of SI [2, 7, 8]. The GMC consider reflective practice as a vital attribute contributing to improving patient care and specifically request that doctors:

*‘reflect on your practice and your approach to medicine, reflect on the supporting information you have gathered and what that information demonstrates about your practice identify areas of practice where you could make improvements or undertake further development demonstrate that you are up to date and fit to practise’ .****GMC (2013)***

Compelling evidence for the impact of reflection on actual performance is currently absent [9]. There has been some exploration of the use of reflection within portfolios with some encouraging results for learning [10] but a requirement to provide SI or maintain a portfolio does not automatically mean that reflection will occur [11]. There is also a wider debate about the assessment of evidence in portfolios and in particular whether reflective writing can and should be assessed [6, 10]. Dohn suggests that reflection, a cognitive process and its correlation with actual practice is complex and non-linear [12]. Making surrogate artefacts which are supposed to represent ‘actual’ practice meaningful and educational has been challenging in other settings such as postgraduate training with a widespread concern that medical education and professional development has become simply a tick-box exercise [13]. Dohn goes further in her criticism of reflective practice. She suggests that an individual’s act of reflection permits a singular reconstruction of reality which tends to proffer competence and conformity. Reflection, being a mental process, she argues can undermine professional development by distancing the practitioner from the actual actions of practice. Reflection, simply demonstrates the ability to talk and write about practice, and is a skill not necessarily linked with competence. Competence she forwards is demonstrated through action *in* the workplace [12].

The theoretical underpinning of the term ‘reflection’ is not defined by the regulator, neither is a standard or an agreed way of assessing reflection set out for the appraiser and RO by the GMC. There is very limited literature about the use, and in particular the assessment of reflection for revalidation purposes and furthermore, concerns exist about the robustness of SI when used in appraisal and revalidation [14]. Threats to reliability and validity of SI are listed in Table 2.

**Table 2:** Threats to reliability and validity of SI in revalidation

• The ability of the appraisee to ‘cherry-pick’ favourable SI • The subjective and inconsistent interpretation of SI by appraisers • The subjective and inconsistent interpretation by appraisees • Access to SI, for example patient feedback, which may not be ready available for some medical professionals • Some SI may be important to certain medical professionals, for example outcome data for surgical specialties, currently not mandatory • The administration of MSF • ‘Assessing’ the quality of reflection	

Revalidation requires the RO to make a positive recommendation that a doctor is fit to practice; a complex question based on a third-party judgment of past performance centered largely on appraisal documentation within a portfolio. When assessing complex competencies, the sum of an assessment process must be greater than its individual parts and qualitative sources are required. Provided there is adequate sampling across domains, even a seemingly subjective assessment method can be valid [15]. The portfolio of SI required for revalidation therefore aims to sample the whole scope of practice with no single portion taken in isolation [16].

Some areas of postgraduate training in the UK have cautiously moved the focus of SI towards formative assessment [17]. Revalidation has therefore refreshed the debate about how to use portfolios, the SI and reflective statements as evidence for summative decisions, as part of a wider reflective learning tool or, controversially, as both [18].

### Background to the study

Each UK healthcare organisation has a dedicated Responsible Officer (RO), most commonly a medical director, responsible for the revalidation recommendation of the doctors within that organisation. Themselves doctors, ROs undergo revalidation with their recommendation made following an appraisal interview conducted by an experienced appraiser and authorised by a higher level RO, in London a ‘regional medical director’ who makes supplementary checks on SI.

As part of a wider evaluation of the NHS England London region’s approach to RO revalidation, [19] this study aimed to explore perceptions of both ROs and their appraisers about the strengths and weaknesses of using SI in revalidation appraisal, a summative assessment.

The research questions for the ROs were: what is the perceived acceptability and feasibility of the role of the portfolio and supporting information required for revalidation and of the reflective process within this. For appraisers the research questions were: what are the perceptions of appraisers of the sufficiency of supporting information provided, the quality of the SI provided, and of the requirement to make judgements on the basis of this information. The aim of this research was to inform future iterations of revalidation.

This study specifically explores SI from the perspective of the appraiser, often neglected in previous studies [20, 21]. This was a unique opportunity to access a cadre of highly experienced appraisers and their appraisees at the forefront of revalidation; a group not only able to report their own experiences about SI in appraisal but meaningfully reflect on how the process may affect the doctors under their remit. Although not representative of the majority of doctors collating SI and undergoing revalidation, these proponents of revalidation offer a unique perspective, are used to making high-consequence decisions and are pioneers in revalidation who will be shaping future appraisal and revalidation. It is likely that challenges experienced by this group of senior clinicians in managerial roles are only likely to be magnified for others.

## Methods

This qualitative study undertaken in late 2013 after the first round of revalidation used focus groups and one-to-one interviews to explore the experiences of ROs and RO appraisers affiliated to NHS England London region of using SI in revalidation appraisal.

*RO Appraisers:* Focus groups are a means of bringing together of a specific set of people to have a conversation centred on the research problem, its ‘goal …to create a candid, normal conversation that addresses, in depth, the selected topic’ [22]. They are an efficient means of generating a wide range of information through social interaction. In particular, focus group discussions were chosen in order to explore individual perspectives but to also allow participants to compare and contrast their views and experiences within the groups. Having only RO appraisers in the groups and limiting the size of groups to five to six participants created an environment where participants’ stories could be heard and explored in detail [23]. Such ‘focused interviews’ allowed a degree of control by both the researcher with their list of enquiries, and with the participants in the way they choose to answer them [24].

Email invitations were sent to all RO appraisers in the NHS England London region. Focus groups were timetabled to coincide with an appraiser training day with two additional dates offered for those unable to attend. One RO appraiser was unable to make any dates and was interviewed separately. Focus groups were facilitated by a senior researcher with an observer making notes. Seventeen of 24 of the NHS England London region RO appraisers participated via three focus groups (each with five or six participants) and one interview.

*ROs:* One-to-one interviews were the chosen methodology for gathering data from the ROs. One-to-one interviews were chosen to capture the views and experiences of the RO’s about their RO appraisal. Interviews, rather than questionnaires, were considered more appropriate in providing a more comprehensive picture of the issues involved in the appraisal process. The qualitative interview encourages data generation through participants talking about their own experience. ROs in this study came to this new role with a diverse range of previous experiences and capabilities and were working in a diverse range of organizational settings. Therefore one to one interviews encouraged them to tell their stories, and share their unique experiences and reflections as well as address the prompts in the interview schedule. One-to-one interviews were important to provide ROs the privacy to share sensitive issues that may not have been raised in group settings.

ROs represented a population with a varied experience of professional appraisal [25] and were purposively sampled to represent the diverse range of organisations as classified by an organizational typology (Table 3). The initial aim of the study was to interview 10–15 % of the NHS England London region ROs. This number increased as data saturation did not occur in the independent sector – particularly the smaller organisations, who lacked of uniformity of experience. All NHS England London region ROs were e-mailed and asked to opt out if they did not wish to be contacted about this study. No ROs opted out. Interviews were conducted via telephone or at a location convenient for the RO. Two e-mail reminders were sent. 28/124 ROs were interviewed and their organisational typology are included in Table 3.

**Table 3:** Organisation classification of the designated bodies and RO sample stratification

Organisation classification	Number of ROs	Number of ROs invited	Number of participants interviewed
NHS Foundation Trust	16	6	4
NHS Trust	27	9	6
NHS Area Teams (for general practice)	3	3	3
Independent – Large^1^	26	4	4
Independent – Small^2^	43	18	7
Independent – Other^3^	26	7	4
Total	140	47	28

^1^> 50 employees

^2^< 50 employees

^3^Includes charities and third sector organisations

Semi-structured interview schedules were devised for RO interviews and a question schedule for appraiser focus groups. Both were piloted before use. All participants consented to take part. Focus groups and interviews were conducted by one of six researchers (AG, DG, DF, AV, CO, LJS), and were recorded and transcribed by a professional stenographer. Data were anonymised and managed using QSR NVivo 10© [26]. Initial data analysis and field notes were used to inform analysis.

RO and RO appraiser data were analysed independently to fully explore themes within each data set before being compared. Thematic data analysis was inductive, with meaning flowing from the data to form themes and concepts. It was also deductive and explored issues posed by the research questions around collecting and curating SI, including mandatory items in the minimum dataset, and making judgements [27]. Each data set was analysed independently by four researchers who created initial coding themes and subthemes. Themes were discussed, refined and agreed at research progress meetings before agreement on a final coding framework was reached. Once no new themes were identified, the final coding framework was applied to the whole dataset.

This study was assessed as a service evaluation by UCL Joint Research and Ethics Committee Office. This study was funded by NHS London England.

## Results

Four overarching themes were identified in relation to supporting information or evidence: *quantity versus quality*, *patient and colleague MSF*, *interpreting SI*, *reflection and learning.*

### Quality vs. quantity

Many ROs reported it was relatively simple to gather SI. Being in a senior role was an asset as they could access documentation that was needed, or had staff who could gather it for them. Despite this, gathering and categorising SI was reported to be a time consuming process. ROs from smaller organisations reported difficulty in gathering certain pieces of information. Three ROs relayed stories about having to gather additional SI about their full scope of practice which were small areas or roles of practice, getting affidavits or letters of commendation from other organisations before they were recommended for revalidation by their appraiser.

One common theme centred on quality versus quantity. There was a lack of clarity about how much SI was required and what constituted ‘acceptable’ quality. ROs discussed strategies which varied significantly from uploading everything they could find, to being tactical about what they presented. There was concern about getting the right balance, and many ROs mentioned how they would improve their choice of evidence next time round:

*So I think that was the first sort of ‘Oh my God’ moment in this…you know what does one actually select. There’s a massive amount of paperwork that goes through your hands, what’s relevant, what’s irrelevant.*

RO1

* didn’t have a very clear view [of the SI required]. I responded to that by providing everything I could possibly find… It was a safe way of proceeding, it wasn’t inadequate, but the comment was made was perhaps we don’t need quite so much next time.*

RO25

A few ROs talked about being more strategic, picking a single piece of SI that represented the quality of their performance; one had strategically matched SI to the domains of GMP and uploaded information to justify their revalidation in that context. This approach was felt to give the appraiser a better purchase on the ROs key roles and capabilities:

*I had a list of evidence that was an arm’s length long. And when I actually started to populate the system and started to apply my evidence base opposite good medical practice as it’s defined within the system, I found I needed about 20 % of my evidence base.*

RO28

RO appraisers commented on the ‘avalanche’ of SI frequently provided, which made the pre-appraisal process unnecessarily long. Typically 30 pieces or SI were produced, and in the extreme 49 documents. Sifting through these was deemed important but RO appraisers valued a checklist about the minimum SI data set required for revalidation produced during the course of this study. They wished that it had been distributed to the ROs for their information.

### Multi-source feedback *(MSF)*

The study was interested in all forms of SI however, MSF from colleagues and patients dominated discussions by RO as well as their appraisers. Receiving colleague MSF was routine for most ROs, who found receiving results and discussing these during the appraisal unproblematic. However, appraisers often found it difficult to assess colleague MSF (particularly negative comments) because they were unfamiliar with the ROs’ organisations. In the light of negative results from 360 MSF it was the RO’s reaction that was considered as critical but RO appraisers suggested that this sort of SI would be more usefully discussed with someone from the ROs’ own organisation so it could be understood in context:

*But I’m wondering whether the process should be that they have their debrief on the 360 with the somebody, that might be independent or might be in their organisation, and they then bring their 360 and their reflections on it to the appraisal itself.*

#### Focus group 1

Indeed, one RO had already made a deliberate decision to discuss the MSF with a colleague rather than his appraiser:

*I chose them rather than my appraiser, because that person was quite a senior colleague and they understand my work and understand the organisation, so therefore could give very good constructive feedback, which is what they did.*

R021

Patient feedback caused particular difficulty for some ROs. Some were not in clinical practice, had little or no patient contact, or were in specialties which were unable to provide personalised patient multisource feedback:

*One of my doctors is a child psychiatrist who is involved in a lot of child protection work, so is very concerned about getting patient feedback from children who’ve been taken into care*

#### Focus group 1

One RO questioned the validity of patient feedback as a form of SI for revalidation:

*In principle it’s a good idea, but I have a major concern for, for example, the patient questionnaires… I don’t think it’s actually valid, because if you wanted you could completely fix it - nobody checks who’s handing papers out, nobody checks who’s collecting them in … I think the patient feedback thing is fundamentally flawed.*

RO27

### Assessing SI

There was discussion amongst RO appraisers and ROs about the composition of SI and how this should be used to make judgements. RO appraisers talked about ‘thresholds’ that SI had to show sufficiency and not excellence, and that that was a hard assessment to make. Neither group were really sure what actually constituted high quality SI nor how this should influence their assessment:

*Looking at the evidence, I think there’s a massive loophole in that the five year… what I’m referring to is you’ve only got to do one quality improvement process in five years, and nobody’s actually defined what the standard of that is – it’s up to the appraiser … and it could be a very, very simple audit, the doctor’s reflected on it, it’s a genuine audit, but it could have taken half an hour and that would do for 5 years.*

### Focus group 2

There was confusion in recognising what good practice looked like, particularly in non-NHS organisations.

*The area which is more difficult I found is around the governance of their organisation. Because there’s so many different types of organisation, I can tell what’s good governance in a hospital, but trying to work out what good governance is in a small independent practice is much harder.*

Focus group 2

Secondary checks of SI and appraisal interview outcomes (provided by the Higher level RO) were perceived by most as being a useful safety net but by some as a process in which appraisers were undermined.

*So when NHS London came back with questions or comments, they said well didn’t that undermine and invalidate my role as the appraiser, why are they asking this, you know*

Focus group 1

### Reflection and learning

Appraisers noticed variable levels of reflection on the SI and most commented on the lack of reflective writing. The focus for ROs was on amassing SI and appraisers reported that many ROs had not, on the whole, successfully reflected on material presented. RO appraisers used the appraisal conversation to supplement reflection lacking in the portfolios. Appraisers felt that reflection was almost a separate, often ignored process and this lack of, or delayed reflection led to reduced usefulness of the reflective process and thus the appraisal interview:

*Some of them did avalanche you with material, but very little reflection. And then using the appraisal moment as the reflection opportunity really, cos it’s not in there …I think there’s a real learning curve about reflection. And then using the appraisal moment as the reflection opportunity really…and deciding that that was the place to do it and then commenting on that, rather than us saying ‘Can you send me the reflection of it’ …And for the type of personality that becomes a medical director – the completer finishers, and get on with it and do it and not pause and stop really - I think there’s a reflection element, learning curve for them.”*

#### Focus group 2

ROs supported this view with most discussing their reflection coming from the appraisal interview itself:

*I mean I think we were reflecting and I talked about the reflections that I’ve you know been through in some of the areas. So I mean [appraiser] didn’t say I want to go through every single piece evidence and I want to go through every single reflection on evidence, it was a principle, and I suppose the conversation was about [appraiser] walking away comfortable that I was a sufficiently reflective practitioner, and that my reflection wasn’t airy fairy reflection, it was quite deliberate.*

RO8

## Discussion

### Summary of results

ROs reported that, on the whole, SI required for revalidation appraisal was easy to gather but time-consuming consistent with the findings of the Revalidation Support Team [28]. There were challenges in knowing which documents best represented their own capabilities, how much information to provide, at what level and in which categories to place it. Some ROs reported difficulty in providing certain types of SI dependent on their specific roles or organisations whilst appraisers felt MSF feedback was challenging to deliver meaningfully without contextual knowledge of the organisation.

RO appraisers were concerned about making ‘robust’ judgements about SI, exacerbated when the RO’s strategy had been to provide an over-abundance of data, rather than a considered selection. SI was used in a predominantly prescriptive way, mapped to the minimum dataset rather than being used to develop practice through reflection. Written reflection in these revalidation portfolios was often missing or of poor quality, being largely descriptive. The appraisers explored reflection within the appraisal meeting in order to assess this aspect of professional practice.

### Defining supporting information

Despite some guidance about SI for revalidation from the GMC and other professional bodies, ROs found it challenging to select suitable SI in appropriate quantities. A lack of distinction between what constitutes fitting SI and what does not was evident; any piece of day-to-day paperwork *could* be submitted and may be judged equally as valid as another. This ‘quantity over quality’ approach reportedly diminished the utility of the appraisal reflecting findings from a significant body of similar research, which suggested better guidance on content and quality to reduce unnecessary inclusions [5, 19]. What constitutes ‘quality’ SI is highly problematic, and work defining it is on-going and requiring further research. Future guidance, which is currently being developed, should therefore include content and structure information, quantity recommendations, how it will be assessed. Example documents have been suggested to be of benefit and could be annotated in accordance with assessment criteria [29].

Some ROs, particularly those working in small independent organisations, had difficulty providing the minimum data set. Similar problems may impact on other doctors and certain protected characteristics for example, women on maternity leave and doctors in certain working roles (locums and associate specialists) may find it difficult to meet generic SI requirements [30]. This issue is fundamental; doctors are a heterogeneous group with varied careers working in different settings and roles. This diversity is likely to affect the acceptability and their ability to provide SI in different areas and adaptable, increasingly individualised systems may well be needed.

### Evaluating supporting information

Decision making and making objective judgements about SI poses significant challenges. In General Practice, appraisers have repeatedly identified their unease about this aspect of appraisal and the findings from this study were no different [31]. Being confident to assess SI in the context of a summative assessment is particularly challenging [5, 32]. The assessment of professional practice portfolios is arguably more complex than the assessment of those in medical education and training, especially as in these setting portfolios are not the sole means for ensuring competence. Professional practice and CPD is highly individualised, without an agreed curriculum or learning objectives; therefore there is an even greater limit to which portfolios can be standardised. That combined with a current lack of clear quality descriptors explains why appraisers in this study sometimes found it hard to judge whether information provided was robust-enough. Whether or not non-standardised portfolios can be assessed, especially when some SI is subjective and highly unique is an area of debate [33].

There are also limitations of one appraiser, in one face-to-face setting, making decisions that will support revalidation recommendations. Murphy and colleagues, using a common set of SI, suggested that four independent scrutinisers were needed to provide a reliable recommendation for revalidation at the level of reliability necessary for high-stakes assessment [8]. Despite this, there is some suggestion that the current form of appraisal is adequately robust to support revalidation [34]. Secondary checks on SI quality performed by the higher level RO left some appraisers feeling undermined, and some ROs concerned they would not be revalidated. Others felt this ‘double-checking’ ensured that the system was robust. A peer-review approach such as this can be acceptable and feasible and it seems important that benchmarking continues with senior level review about SI quality and subsequent revalidation recommendations pending more explicit guidance [25]. This highlights an opportunity for benchmarking to become an educational process as a training activity for appraisers in making judgments.

The revalidation portfolio of SI is unique from most portfolios examined in previous studies, in that it is gathered specifically in preparation for the revalidation interview rather than longitudinally, and it's explicit purpose is for summative assessment. Generalisability of prior findings to this context is therefore limited. Although portfolios have been used in assessment processes within medical education in the past [35], most have a primarily formative goal, and thus tensions arise when also used for summative assessment. Users often complain of a lack of clarity of purpose in portfolios, with a mismatch between conceptualisation and operationalisation [36, 37]. This was not the case in this study in which there was a clearly articulated and understood purpose that was shared by appraisers and appraisees. However, in parallel with other studies there was a disjuncture between the process of gathering and selecting evidence, and reflection on the learning that had occurred during acquisition of that evidence. A common theme in research on portfolio use within the professions is that reflection is enhanced by a mentor (in this case the appraiser) [6, 37, 38]. As the cornerstone of revalidation, making objective judgements about SI is an area where a great deal more research is required, in particular analysing the attributes of ‘quality evidence’ to develop level descriptors.

Perhaps alarmingly, the only evidence-based SI used in revalidation appraisal is colleague MSF and benchmarking is likely to be equally helpful in making judgements about this [39]. MSF reduces bias and increases objectivity by using multiple raters [40]. Delivering colleague MSF results during the appraisal interview was not a familiar practice for most RO appraisers. Unfamiliarity with the RO, their organisation and the sometimes sensitive nature of the information, coupled with a feeling that they could have been better trained for this aspect of the role made it challenging. By comparison, in business, MSF is most effective in creating improvement when facilitated by organisations themselves, rather than external facilitators [41].

### Learning and reflection

The use of portfolios in medical education is expanding along with literature to support their use in continuing professional development, learning and increasingly, assessment [15, 37]. It is accepted that they will remain integral to learning and assessment in medicine for the foreseeable future. In accordance with revalidation as a mechanism to protect the public, presently the role of SI within the portfolio relates predominantly to one facilitating assessment; its value as a learning tool as yet, has not been fully realised in this context, consistent with current literature which suggests there is still considerable scepticism [42].

GMC guidance maps SI requirements to GMP and suggests doctors should reflect on their own clinical practice in relation to this framework. So concerned with delivering the SI required to meet revalidation requirements, ROs delayed learning and reflection until the appraisal interview, where it was facilitated by the appraiser. In this context, reflection and learning were described favourably. This is important, affirming that SI is an important developmental tool, recognised as such by appraisees, and that there does exist a place for reflection in the process. However, there are two important caveats. The revalidation portfolio is required to contain reflective writing but there is considerable controversy as to whether reflection can and should be assessed. In the context of summative assessment, as highlighted in the results, there may be a temptation to ‘construct’ a picture of a competent professional through both choice of SI and reflection. Whether reflection does have the capacity to improve clinical practice is still unproven. Reflection as an individual act removed from practice may do little to improve *actual* performance and needs greater empirical investigation [12].

The revalidation portfolio seems to significantly shift the emphasis of SI from learning to assessment, reflecting the current environment in medical education focused on outcomes and competence acquisition as well as the political climate in terms of patient and public engagement [43]. The focus currently sits away from the individual learner and their needs and close to what is required of doctors by today’s society. A prescribed reflective element may be beneficial but proceeded to with caution given the mixed attitudes towards reflective practice [44]. The cautious purposeful facilitation of reflective practice in revalidation portfolios requires careful evaluation and implementation. Equally, a move towards workplace based assessments is unlikely to be well received by senior doctors but peer-review may well play an important role. Focus on developing and refining the process to ensure the balance between learning and assessment is optimised is a key focus for the medical profession

There are several limitations of this study. This study explored the views of a very specific group of senior doctors undergoing, responsible for revalidation undergoing their own revalidation and their very experienced appraisers. As such both groups will have a vested interest in revalidation itself which will almost certainly skew the results, in fact this group, as proponents are recognised to have a more positive view of revalidation [19]. Despite undergoing a magnified process, these doctors have attempted this process first, and will be acutely aware that the challenges they faced may be reflected onto the general population of doctors revalidating and may have some influence on further iterations of revalidation, providing a significant relevance to these findings. The small scale and single location (London) of the study reduces the generalisability of the results. Additionally, its participatory nature may mean that some views were over or under-represented or missed. Small independent sector organisations were unique in their heterogeneity; they included small private healthcare companies and charities and recruitment of ROs from this typology was more difficult. The nature of qualitative data may in itself be subject to bias in the collection, analysis and interpretation of data itself. To mitigate this, researchers worked independently and then together in collating and coding data and ran an inter-coder analysis to show agreement and refine areas of non-agreement.

## Conclusion

There is a significant literature which supports the use of portfolios in promoting learning, largely through reflective practice and in the context of formative assessments. There is less research in the use of portfolios in assessing professional competence and controversy as to whether reflection can and should be assessed, particularly with reference to this novel process of revalidation. SI provides the basis for the ‘evidence’ of a doctor’s fitness to practise medicine but there is little empirical evidence as yet to support the validity and reliability of SI in ensuring improved patient care and impact on a doctors’ performance. Research is needed to clarify what SI is the most meaningful and offers a true reflection of actual practice, what constitutes quality, how it should be judged and evaluated and what its role is in learning. Those responsible for appraisal and revalidation must strive to develop clear evidence-based guidelines detailing expectations for content and reference criteria for making judgments. Further research is needed evaluating what constitutes high-quality supporting information and how it should be used in assessment. The utility of SI in reflective learning and professional development must be harnessed and not left redundant, ever displaced by assessment.

## Abbreviations

GMC: General medical council
GMP: Good medical practice
MSF: Multi source feedback
NHS: National health service
RO: Responsible officer
SI: Supporting information
UCL: University College London
UK: United Kingdom
